# Gamma Irradiation-Induced Preparation of Graphene–Ni Nanocomposites with Efficient Electromagnetic Wave Absorption

**DOI:** 10.3390/ma11112145

**Published:** 2018-10-31

**Authors:** Youwei Zhang, Hui-Ling Ma, Ke Cao, Liancai Wang, Xinmiao Zeng, Xiuqin Zhang, Lihua He, Pinggui Liu, Zhiyong Wang, Maolin Zhai

**Affiliations:** 1 Aviation Key Laboratory of Science and Technology on Stealth Materials, Beijing Institute of Aeronautical Materials, Beijing 100095, China; ywzhang_pku@163.com (Y.Z.); clyzxq@bift.edu.cn (X.Z.); 2 Beijing Key Laboratory of Clothing Materials R&D and Assessment, School of Materials Science & Engineering, Beijing Institute of Fashion Technology, Beijing 100029, China; helihua-xjt@163.com (L.H.); liupinggui@hotmail.com (P.L.); zywang910@163.com (Z.W.); 3 Beijing Key Laboratory of Radiation Advanced Materials, Beijing Research Center for Radiation Application, Beijing 100015, China; ckcoco2014@163.com (K.C.); 13910127876@139.com (L.W.); sherry_0282_cn@sina.com (X.Z.); 4 Beijing National Laboratory for Molecular Sciences, Department of Applied Chemistry and the Key Laboratory of Polymer Chemistry and Physics of the Ministry of Education, College of Chemistry and Molecular Engineering, Peking University, Beijing 100871, China

**Keywords:** graphene oxide, Ni nanoparticles, gamma-ray irradiation, radiation-induced reduction, electromagnetic wave absorbing

## Abstract

A facile and environmentally friendly method is proposed to prepare reduced graphene oxide–nickel (RGO–Ni) nanocomposites using γ-ray irradiation. Graphene oxide (GO) and Ni^2+^ are reduced by the electrons which originated from the gamma radiolysis of H_2_O. The structure and morphology of the obtained RGO–Ni nanocomposites were analyzed using X-ray diffraction (XRD) and Raman spectroscopy. The results show that Ni nanoparticles were dispersed uniformly on the surface of the RGO nanosheets. As expected, the combination of RGO nanosheets and Ni nanoparticles improved the electromagnetic wave absorption because of the better impedance matching. RGO–Ni nanocomposites exhibited efficient electromagnetic wave absorption performance. The minimum reflection loss (RL) of RGO–Ni reached −24.8 dB, and the highest effective absorption bandwidth was up to 6.9 GHz (RL < −10 dB) with a layer thickness of 9 mm.

## 1. Introduction

Electromagnetic radiation is considered as a serious pollutant due to the rapid growth of electronic and communication technology, which would be a potential threat for human health [1,2,3]. An effective method of solving these problems is using electromagnetic wave (EMW)-absorbing materials to absorb those unwanted electromagnetic energies [4,5]. Many efforts were made to prepare EMW-absorbing materials with low density, a broad absorption frequency band, high absorption capacity, and good thermal stability, for meeting military purposes and improving the human living environment [6,7].

Traditionally, metallic magnetic metals including carbonyl iron, Fe, Co, Ni, and their alloys are widely used as EMW-absorbing materials because of their high complex permeability [8,9,10,11]. However, their heavy weight and poor antioxidation limit their application in military and industry fields. Therefore, EMW-absorbing materials with low density and strong EMW absorption characteristics are urgently required.

It was reported that carbon nanotubes, graphene, and carbon foam exhibit many satisfactory characteristics, including light weight, superior corrosion resistance, and high dielectric loss [12,13]. Among these materials, reduced graphene oxide (RGO) may be a potential material for EMW absorption because the residual oxygen-containing groups and structure defects of RGO can induce polarization relaxation. Furthermore, RGO promotes the electronic energy level from the continuous state to the Fermi energy level [14]. However, pristine RGO mainly possesses dielectric loss, along with extremely low magnetic loss, which give rises to small electromagnetic impedance matching and low absorption properties [15]. Therefore, nanocomposites consisting of RGO and magnetic materials were exploited to satisfy the impedance matching condition and improve the microwave absorption capability [16]. For example, Tian et al. fabricated two-dimensional functional alpha-Fe_2_O_3_/RGO nanocomposites through a green and controllable hydrothermal method. The minimal reflection loss reached −61.0 dB at 12.0 GHz when the thickness of the resulting nanocomposites was only 2.5 mm [17]. Long et al. prepared jellylike cylinder graphene/Mn_3_O_4_ composites via a solvothermal process. The obtained composites exhibited a minimal reflection loss of −14.2 dB from 2 to 18 GHz [18]. However, these materials were prepared via solvothermal [19,20], hydrothermal [16,21], chemical reduction [22], and chemical vapor deposition (CVD) methods [23], which are more energy-intensive, and use toxic reagents or a complicated fabrication process. Therefore, a green and facile method of preparing magnetic nanoparticle/RGO nanocomposites at ambient temperature is urgently required.

Gamma-ray irradiation is an attractive, facile, and clean method of preparing metal-nanoparticle-loaded graphene nanocomposites. In our previous work, graphene nanosheets decorated with Ag nanoparticles [24] and Pt nanoparticles [25] were prepared using gamma-ray irradiation-induced reduction at ambient temperature. These results suggest that magnetic nanoparticle/RGO hybrids can be prepared using the radiation-induced simultaneous reduction of graphene oxide (GO) and metal ion precursors.

In view of its high Snoek’s limit, nickel is considered as a potential EMW-absorbing material for high frequencies over the gigahertz range. Therefore, in the present study, Ni nanoparticle-decorated RGO nanocomposites were prepared via a one-step gamma-ray irradiation-induced reduction. The structures of the obtained hybrids were analyzed using X-ray diffraction (XRD) and Raman spectroscopy. The influences of dose and dose rate on the permeability, permittivity, and EMW absorption performance were also explored. The combination of the magnetic loss of Ni nanoparticles and the dielectric loss of RGO would benefit the enhancement of EMW absorption performance.

## 2. Materials and Methods

### 2.1. Materials

GO was bought from The Sixth Element (Changzhou) Materials Technology Co., Ltd. (Changzhou, China). Nickel acetate (Ni(CH_3_COO)_2_·4H_2_O) was supplied by Tianjin Fuchen Chemical Reagent Co. Ltd. (Tianjin, China). All other chemicals were analytical grade and were obtained from Beijing Chemical Factory (Beijing, China).

### 2.2. Preparation of RGO–Ni Materials

GO (100 mg) was dispersed in 50 mL of deionized water. The solution was treated for 1 h in an ultrasonicator (Scientz-II, China). Then, an appropriate amount of nickel acetate aqueous solution was added to the solution and stirred for a further 20 min. The mixture was deaerated with nitrogen bubbling for 30 min, and was subsequently irradiated in a ^60^Co source (Peking University) at doses of 50 kGy and 200 kGy. After the irradiation, the mixture was filtrated through a 0.45-μm polypropylene membrane and washed with ethanol. The products were vacuum-dried at 60 °C overnight. The RGO–Ni composites prepared at 50 kGy and 200 kGy were named RGO–Ni-50 and RGO–Ni-200, respectively. Upon varying the absorption dose, both RGO–Ni composites were subjected to further electromagnetic parameter measurements.

### 2.3. Characterizations

The crystal structures of the products were identified using an X-ray diffractometer (XRD) with Cu Kα radiation (Rigaku D/MAXRC). Raman spectra were recorded with a confocal Raman spectrometer system (In Via, Renishaw, Gloucestershire, UK) at an excitation wavelength of 514.5 nm. The accuracy of the Raman and XRD measurements was 5%. The samples used for complex permittivity and permeability measurements were prepared by dispersing composites into paraffin at a mass fraction of 20%. The obtained sample was subsequently pressed into a ring (outer diameter (D_outer_) = 7.0 mm and inner diameter (D_in_) = 3.04 mm). The electromagnetic parameters of the samples from 2 to 18 GHz were measured in accordance with standard SJ-20512-1995 (test methods for permittivity and permeability of micowave high-loss solid materials) using an Agilent N5230C network analyzer. The test error was less than 10%.

## 3. Results and Discussion

**Figure 1:**
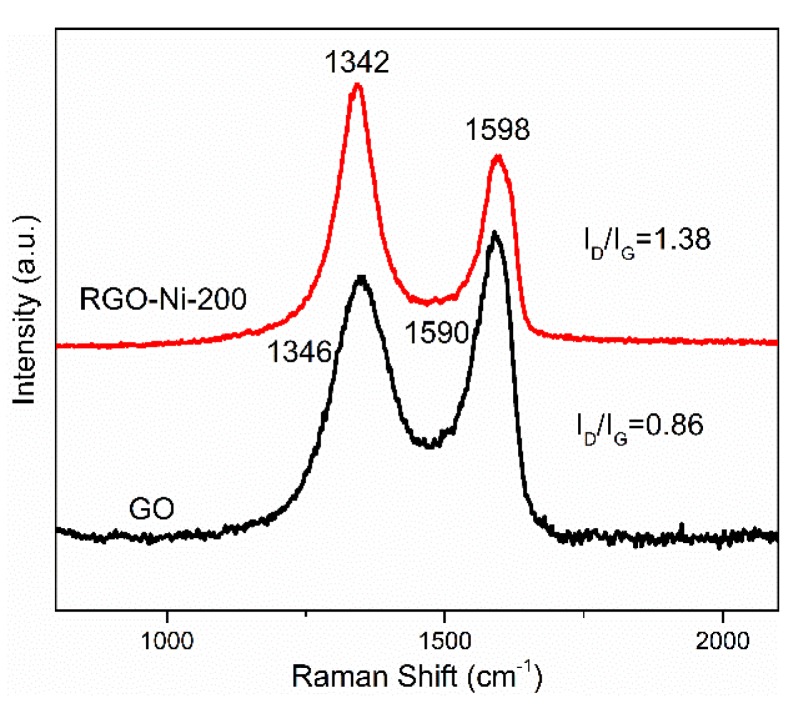
Raman spectra of graphene oxide (GO) and reduced GO/nickel composite irradiated at 200 kGy (RGO–Ni-200).

Raman spectroscopy is an effective method for resolving the bonding state of carbon atoms and the changes in graphitic structure of carbon materials during the chemical process. The Raman spectra of GO and RGO–Ni are shown in Figure 1. There are two peaks located at around 1346 cm^−1^ and 1590 cm^−1^, corresponding to the D band (related to the in-plane vibration of the *sp*^2^ carbon atoms in a two-dimensional (2D) hexagonal lattice) and the G band (associated with vibration of the *sp*^3^ carbon atoms of disordered graphitic carbon) of carbon-based materials, respectively. The ratio of D and G band intensity (I_D_/I_G_) is an indicator for evaluating the quality of carbon materials. The I_D_/I_G_ ratio of RGO–Ni increased to 1.38, higher than that of GO (0.86), suggesting more defects and smaller *sp*^2^ domains were created on RGO–Ni during the reduction of GO and the attachment of Ni particles. The full width at half maxima (FWHM) of the G peak for GO and RGO–Ni were 85 cm^−1^ and 80 cm^−1^, respectively. The load of Ni nanoparticles and the restoration of the *sp*^2^ network of the carbon-bonded structure both contributed to the slightly smaller FWHM for RGO–Ni. Appropriate levels of defects could improve the attenuation of incident EMW, because the enhancement of dipole polarization relaxation at the sites of defects can induce the dielectric loss of electromagnetic energies.

**Figure 2:**
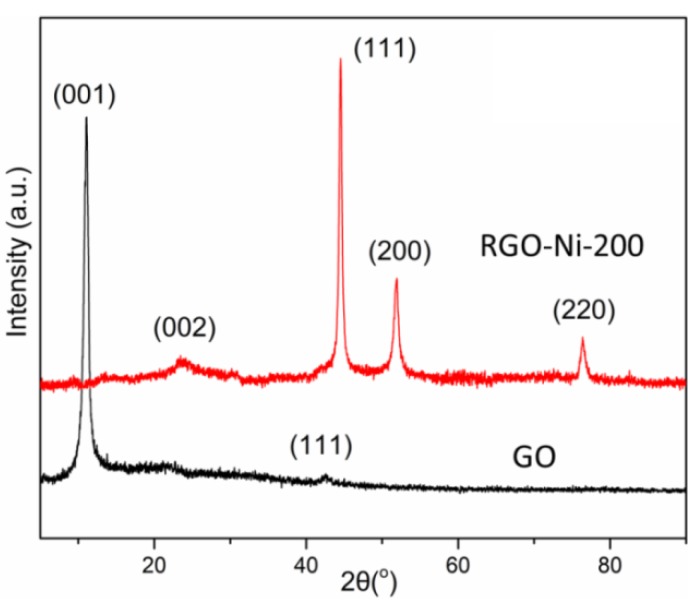
X-ray diffraction (XRD) patterns of GO and RGO–Ni-200.

The XRD spectra of GO and RGO–Ni are presented in Figure 2 to analyze the crystalline properties of the materials. The characteristic diffraction peak of GO appeared at 2θ = 11.2°, which suggested an interlayer distance of 0.79 nm. For RGO–Ni, the diffraction peaks appeared at 76.4°, 51.9°, and 44.5°, corresponding to the (220), (200), and (111) planes of face-centered cubic-structure nickel, respectively. The diffraction peaks were sharp, reflecting the high crystallinity of the Ni nanoparticles. Furthermore, the weak and broad reflection peak located at 2θ = 23.3° was assigned to the (002) plane of RGO–Ni, indicating the restoration of the *sp*^2^ network of the carbon-bonded structure following the reduction of GO by γ-ray radiation. As described in our previous work, GO can be reduced by γ-ray radiation in a solution of dimethylformamide (DMF) or water [26,27]. Therefore, it was concluded that well-crystallized Ni nanoparticles were formed on the surface of the RGO nanosheets.

**Figure 3:**
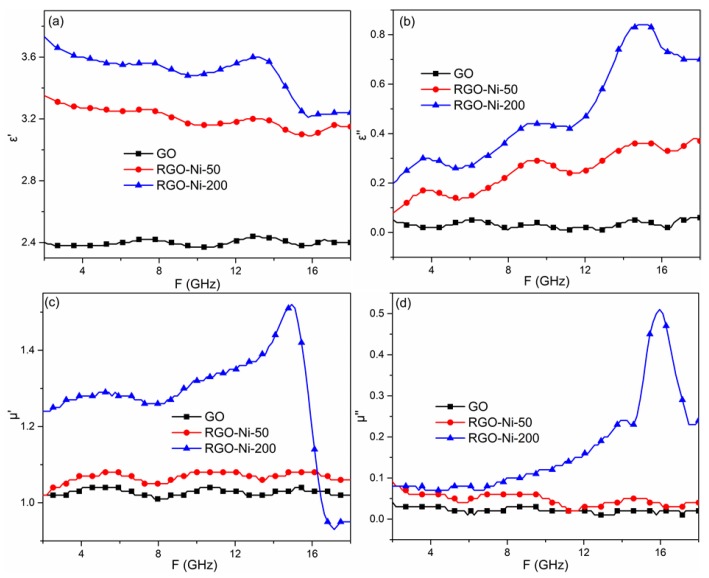
(**a**,**b**) Complex permittivity (ε′, ε′′) and (**c**,**d**) complex permeability (μ′, μ′′) spectra of GO, RGO–Ni-50, and RGO–Ni-200 vs. frequency within the range of 2–18 GHz.

The relative complex permittivity (ε_r_ = ε′ − jε′′) and complex permeability (μ_r_ = μ′ − jμ′′) spectra of GO, RGO–Ni-50, and RGO–Ni-200 vs. frequency within the range of 2–18 GHz are shown in Figure 3. The values of ε′ and μ′ are related to the energy storage capability. The values of ε′′ and μ′′ represent energy dissipation, which can be ascribed to the conduction, resonance conduction, and relaxation mechanisms [28]. GO has the lowest value of ε′ because of its disordered structure. The values of ε′ for RGO–Ni-50 and RGO–Ni-200 were much higher than that for GO, which decreased from 3.38 at 2 GHz to 3.10 at 16 GHz, and from 3.79 at 2 GHz to 3.25 at 15.8 GHz, respectively. The larger values of ε′ were attributed to the excellent electric conductivity of RGO–Ni-50 and RGO–Ni-200 following the γ-ray induced reduction of GO. The imaginary region (ε′′) of permittivity is shown in Figure 3b. The value of ε′′ for GO was constantly zero between 2 and 18 GHz. For RGO–Ni-50 and RGO–Ni-200, there was an increase in the value of ε′′ across the whole frequency range. Obviously, values of ε′′ for RGO–Ni-200 were higher than those for RGO–Ni-50.

Based on the free electron theory, ε′′ ≈ 1/2πε_0_ρf, where ε_0_ is the vacuum permittivity, f is the frequency of the electromagnetic wave, and ρ is the resistivity [29]. The conductivity of RGO–Ni-200 was much higher than that of RGO–Ni-50, leading to a decrease in resistivity and an increase in ε”. The permeability (μ′, μ′′) is shown in Figure 3c,d. The values of μ′ and μ′′ for GO were in the vicinity of 1.0–1.1 and 0–0.1, respectively. RGO–Ni-50 had the same values as GO, which was attributed to the lower content of Ni particles absorbed onto the surface of RGO at the lower irradiation dose. As for RGO–Ni-200, higher permeability (μ′, μ′′) values were observed from 12.0 to 17.0 GHz compared to those seen for GO and RGO–Ni-50, due to the magnetic properties of the increased number of Ni particles [30].

**Figure 4:**
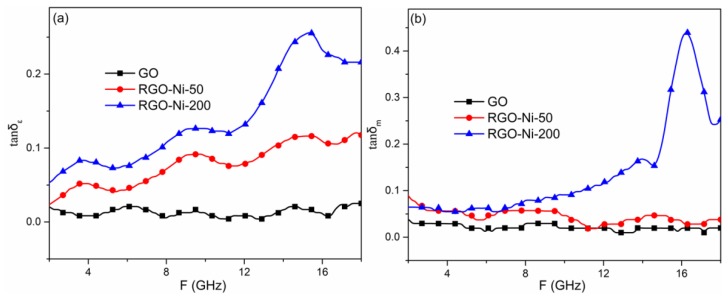
The corresponding dielectric loss tangents (**a**) and magnetic loss tangents (**b**) of GO, RGO–Ni-50, and RGO–Ni-200.

The electromagnetic loss capabilities of the as-prepared products were estimated from the loss tangent. Figure 4 shows the dielectric constant tangent (tanδε = ε′′/ε′) and magnetic loss tangent (tan δm = μ′′/μ′) spectra of GO, RGO–Ni-50, and RGO–Ni-200 vs. frequency in the range of 2–18 GHz. The dielectric loss tangent values of RGO–Ni-200 were larger than those of GO and RGO–Ni-50, which increased from 0.04 (2 GHz) to 0.25 (15.5 GHz). The magnetic loss tangent values of RGO–Ni-200 were slightly smaller than those of RGO–Ni-50 from 2.0 to 3.5 GHz, before increasing sharply from 0.12 at 12.0 GHz to 0.45 at 16.0 GHz, while the values of GO and RGO–Ni-50 were steady at about 0.05 in the range of 2–18 GHz. These results show that RGO–Ni-200 exhibits much improved dielectric and magnetic loss performance following the introduction of Ni nanoparticles via γ-ray irradiation.

As is well known, an ideal EMW-absorbing material should reduce the reflected loss of EMW at the interface between air and the material. Meanwhile, it should exhibit excellent EMW-absorbing properties within the material. The impedance matching characteristic (*Z*) is very important for minimizing the reflection of electromagnetic waves, and it is expressed using Equation (1).

(1) $$Z=\left[\frac{Z_{in}}{Z_{0}}\right]=\sqrt{\frac{\mu_{r}}{\varepsilon_{r}}}\tan h\left[j\left(\frac{2\pi fd}{c}\right)\sqrt{\mu_{r}\varepsilon_{r}}\right]\approx \sqrt{\frac{\mu_{r}}{\varepsilon_{r}}},$$

where *Z*_0_ is the characteristic impedance of free space, *Z_in_* is the input impedance of the absorber, *f* is the frequency, *d* is the absorber thickness, and *c* is the velocity of electromagnetic waves in free space.

**Figure 5:**
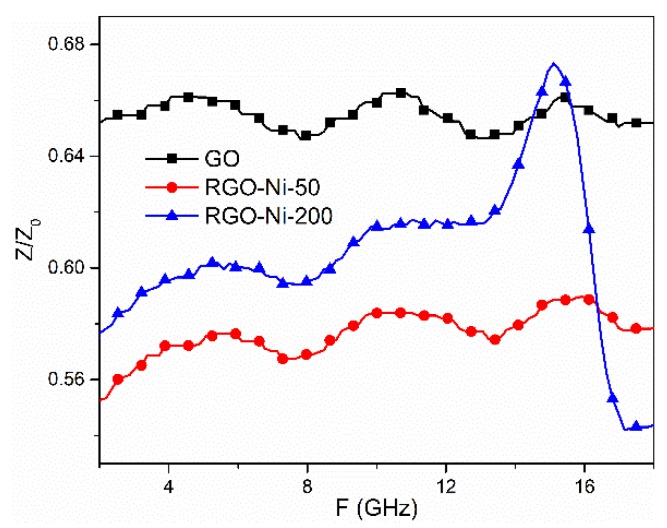
The impedance matching characteristics of GO, RGO–Ni-50, and RGO–Ni-200.

The absorption performances of materials are improved when *Z* is equal or close to 1.0, as almost all of the EMWs incident on the materials change into thermal energy or dissipate through interference [31]. The values of *Z* for the samples at different frequencies are presented in Figure 5. Because of the low permeability and permittivity, the wave absorption of GO was fairly poor, even if the value of *Z* for GO was much closer to 1.0. The values of *Z* for RGO–Ni-200 were much larger than those of RGO–Ni-50 in the frequency range of 2.0 to 16 GHz, suggesting that its characteristic impedance was well matched. Therefore, RGO–Ni-200 may exhibit better EMW absorption properties compared to RGO–Ni-50.

**Figure 6:**
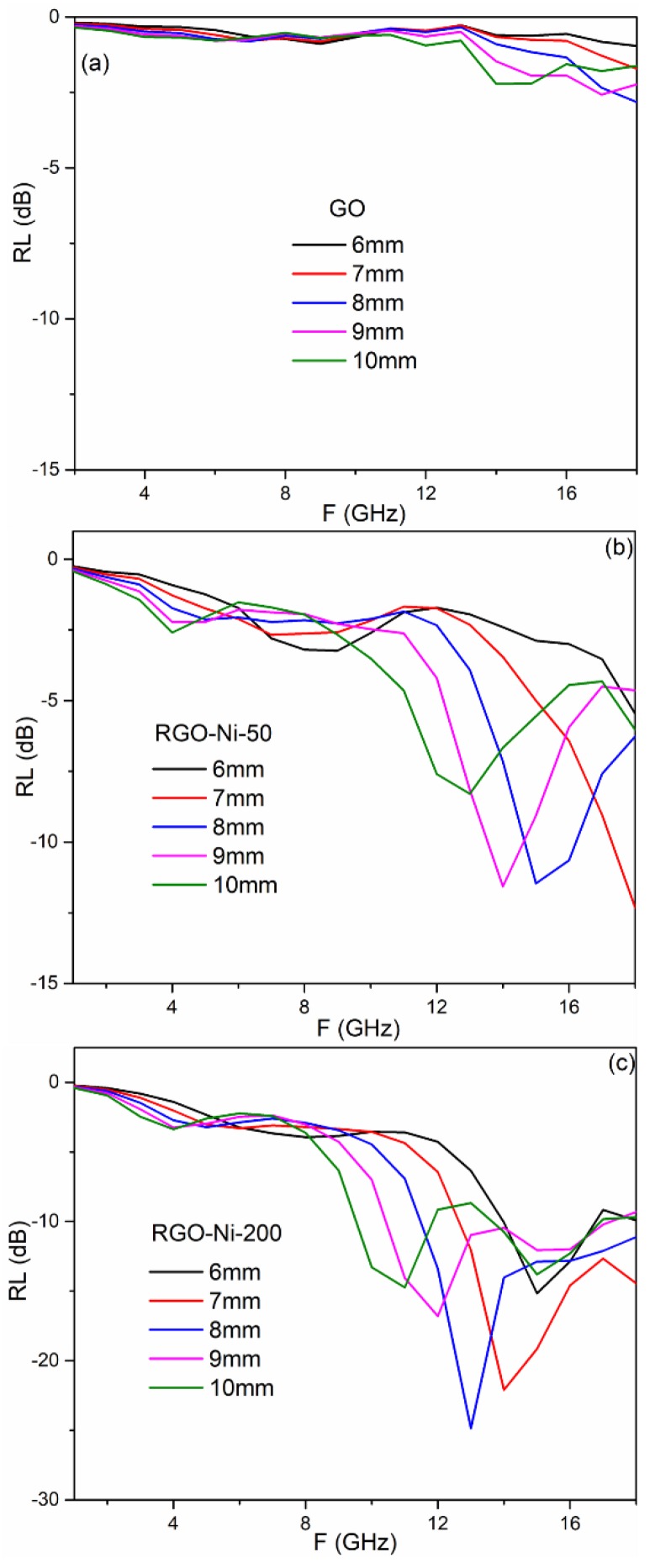
Calculated reflection loss (RL) curves of GO (**a**), RGO–Ni-50 (**b**), and RGO–Ni-200 (**c**) with different thicknesses vs. frequency within the range of 2–18 GHz.

In order to study the electromagnetic absorption performances of the obtained RGO–Ni nanocomposites, the reflection loss (RL) properties of the samples were calculated according to the transmission line theory, using the values of *ε_r_* and *μ_r_* in Equations (2) and (3).

(2) $$RL(dB)=20\mathrm{lg}\left|\frac{Z_{in}-Z_{0}}{Z_{in}+Z_{0}}\right|,$$

(3) $$Z_{in}=Z_{0}\sqrt{\frac{\mu_{r}}{\varepsilon_{r}}}\tan h\left[j\left(\frac{2\pi fd}{c}\right)\sqrt{\mu_{r}\varepsilon_{r}}\right],$$

where *Z*_0_ is the characteristic impedance of free space, *Z_in_* is the input impedance of absorber, *f* is the frequency, *d* is the absorber thickness, and *c* is the velocity of electromagnetic waves in free space. The reflection loss curves of GO, RGO–Ni-50, and RGO–Ni-200 with layer thicknesses of 6–10 mm are presented in Figure 6. The RL values of GO at 6–10 mm were higher than −3 dB across the whole frequency range. Compared to GO, RGO–Ni-50 and RGO–Ni-200 both exhibited enhanced EMW-absorbing performances with RL values below −10 dB at higher frequencies, indicating that 90% of incident microwaves were attenuated. This was probably because the dielectric constant of RGO and the magnetic loss of Ni nanoparticles were complementary. As a higher dose facilitates the formation of Ni nanoparticles and the reduction of GO, RGO–Ni-200 displayed stronger EMW absorption properties in the higher frequency range (10 to 18 GHz). Currently, broadening the effective absorption bandwidth (the frequency range when RL ≤ −10 dB) is still a challenge for EMW-absorbing materials. RGO–Ni-200 with a loading of 20 wt.% presented the highest effective absorption bandwidth of 6.9 GHz when its thickness was 9 mm, covering the frequency range from 10.4 to 17.3 GHz. A maximum RL value of −24.8 dB was obtained at 13.0 GHz when the layer thickness was 9 mm.

EMW attenuation and impedance matching improve the EMW absorption of materials. For RGO–Ni nanocomposites, the two abovementioned factors might be affected by three mechanisms. Firstly, the introduction of Ni nanoparticles (magnetic loss) into the RGO sheets (electric loss) could effectively improve the magnetic loss performances of RGO–Ni nanocomposites. Zhu et al. reported that the main magnetic loss mechanism could be ascribed to natural resonance, eddy current effects, and domain wall resonance [30]. Secondly, Ni nanoparticle-decorated RGO nanosheets might cause a reduction in conductivity and an increase in impedance matching performance [29]. Simultaneously, numerous interfaces and more defects in RGO–Ni would result in interfacial polarization, thereby improving dielectric loss. Finally, there were residual oxygen-containing groups (–C–OH, –COOH) in RGO after gamma-ray irradiation-induced reduction. These residual chemical polar bonds (C–O, C=O) with different electronegativity could act as electric dipoles to increase polarization relaxation [32]. All these factors are favorable for improving EMW adsorption and broadening frequency bandwidth.

## 4. Conclusions

Herein, we demonstrated a facile method of preparing RGO–Ni nanocomposites at ambient temperature. GO and Ni^2+^ were simultaneously reduced using γ-ray radiation. Ni nanoparticles were loaded uniformly onto the surface of RGO sheets. The effective absorption bandwidth of RGO–Ni nanocomposites reached 6.9 GHz (RL < −10 dB) in the range of 10.4 to 17.3 GHz when the thickness was 9 mm. The minimum reflection loss increased to −24.8 dB at 13 GHz. The good EMW-absorbing performance can be attributed to the well-matched characteristic impedance and the complementary nature of dielectric loss and magnetic loss. Therefore, our study suggests that the obtained RGO–Ni nanocomposites with highly efficient absorption properties can be used as a new type of EMW-absorbing materials.

## References

[1] Wu F., Xie A.M., Sun M.X., Wang Y., Wang M.Y. (2015). Reduced graphene oxide (RGO) modified spongelike polypyrrole (PPy) aerogel for excellent electromagnetic absorption. J. Mater. Chem. A.

[2] Ding X., Huang Y., Li S.P., Wang J.G. (2016). Preparation and electromagnetic wave absorption properties of FeNi_3_ nanoalloys generated on graphene-polyaniline nanosheets. RSC. Adv..

[3] Sun X.D., Ma G.Y., Lv X.L., Sui M.X., Li H.B., Wu F., Wang J.J. (2018). Controllable Fabrication of Fe_3_O_4_/ZnO Core-Shell Nanocomposites and Their Electromagnetic Wave Absorption Performance in the 2–18 GHz Frequency Range. Materials.

[4] Zheng X.L., Feng J., Zong Y., Miao H., Hu X.Y., Bai J.T., Li X.H. (2015). Hydrophobic graphene nanosheets decorated by monodispersed superparamagnetic Fe_3_O_4_ nanocrystals as synergistic electromagnetic wave absorbers. J. Mater. Chem. C.

[5] Batrakov K., Kuzhir P., Maksimenko S., Volynets N., Voronovich S., Paddubskaya A., Valusis G., Kaplas T., Svirko Y., Lambin P. (2016). Enhanced microwave-to-terahertz absorption in graphene. Appl. Phys. Lett..

[6] Zhou J., Chen Y.J., Li H., Dugnani R., Du Q., UrRehman H., Kang H.M., Liu H.Z. (2018). Facile synthesis of three-dimensional lightweight nitrogen-doped graphene aerogel with excellent electromagnetic wave absorption properties. J. Mater. Sci..

[7] Zhao T.K., Jin W.B., Ji X.L., Gao J.J., Xiong C.Y., Dang A.L., Li H., Li T.H., Shang S.M., Zhou Z.F. (2017). Preparation and electromagnetic wave absorbing properties of 3D graphene/pine needle-like iron nano-acicular whisker composites. RSC Adv..

[8] Yuan H.R., Yan F., Li C.Y., Zhu C.L., Zhang X.T., Chen Y.J. (2018). Nickel Nanoparticle Encapsulated in Few-Layer Nitrogen-Doped Graphene Supported by Nitrogen-Doped Graphite Sheets as a High-Performance Electromagnetic Wave Absorbing Material. ACS Appl. Mater. Interfaces.

[9] Long Q., Xu Z.Q., Xiao H.H., Xie K.N. (2018). A facile synthesis of a cobalt nanoparticle-graphene nanocomposite with high-performance and triple-band electromagnetic wave absorption properties. RSC Adv..

[10] Wang Y., Wu X.M., Zhang W.Z., Luo C.Y., Li J.H. (2017). Synthesis of ferromagnetic sandwich FeCo@graphene@PPy and enhanced electromagnetic wave absorption properties. J. Magn. Magn. Mater..

[11] Ding X., Huang Y., Wang J.G. (2015). Synthesis of FeNi3 nanocrystals encapsulated in carbon nanospheres/reduced graphene oxide as a light weight electromagnetic wave absorbent. RSC Adv..

[12] Li J.S., Duan Y., Lu W.B., Chou T.W. (2018). Polyaniline-stabilized electromagnetic wave absorption composites of reduced graphene oxide on magnetic carbon nanotube film. Nanotechnology.

[13] Letellier M., Macutkevic J., Kuzhir P., Banys J., Fierro V., Celzard A. (2017). Electromagnetic properties of model vitreous carbon foams. Carbon.

[14] Yan F., Guo D., Zhang S., Li C.Y., Zhu C.L., Zhang X.T., Chen Y.J. (2018). An ultra-small NiFe_2_O_4_ hollow particle/graphene hybrid: Fabrication and electromagnetic wave absorption property. Nanoscale.

[15] Chen T., Qiu J.H., Zhu K.J., Che Y.C., Zhang Y., Zhang J.M., Li H., Wang F., Wang Z.Z. (2014). Enhanced electromagnetic wave absorption properties of polyaniline-coated Fe_3_O_4_/reduced graphene oxide nanocomposites. J. Mater. Sci..

[16] Li J., Zhang D., Qi H., Wang G.M., Tang J.M., Tian G., Liu A.H., Yue H.J., Yu Y., Feng S.H. (2018). Economical synthesis of composites of FeNi alloy nanoparticles evenly dispersed in two-dimensional reduced graphene oxide as thin and effective electromagnetic wave absorbers. R. Soc. Chem. Adv..

[17] Tian Z.S., Dai J., Li J.T., Zhu G.Y., Lu J.F., Xu C.X., Wang Y.Y., Shi Z.L. (2016). Tailored Fabrication of alpha-Fe_2_O_3_ Nanocrystals/Reduced Graphene Oxide Nanocomposites with Excellent Electromagnetic Absorption Property. J. Nanosci. Nanotechnol..

[18] Long Y.T., Xie J.L., Li H., Liu Z.R., Xie Y.H. (2017). Solvothermal synthesis, electromagnetic and electrochemical properties of jellylike cylinder graphene-Mn_3_O_4_ composite with highly coupled effect. J. Solid State Chem..

[19] Singh A.K., Kumar A., Haldar K.K., Gupta V., Singh K. (2018). Lightweight reduced graphene oxide-Fe_3_O_4_ nanoparticle composite in the quest for an excellent electromagnetic interference shielding material. Nanotechnology.

[20] Jin L., Zhao X.M., Xu J.F., Luo Y.Y., Chen D.Q., Chen G.H. (2018). The synergistic effect of a graphene nanoplate/Fe_3_O_4_@BaTiO_3_ hybrid and MWCNTs on enhancing broadband electromagnetic interference shielding performance. RSC Adv..

[21] He J.Z., Wang X.X., Zhang Y.L., Cao M.S. (2016). Small magnetic nanoparticles decorating reduced graphene oxides to tune the electromagnetic attenuation capacity. J. Mater. Chem. C.

[22] Chen T.T., Deng F., Zhu J., Chen C.F., Sun G.B., Ma S.L., Yang X.J. (2012). Hexagonal and cubic Ni nanocrystals grown on graphene: Phase-controlled synthesis, characterization and their enhanced microwave absorption properties. J. Mater. Chem..

[23] Cao Y., Su Q.M., Che R.C., Du G.H., Xu B.S. (2012). One-step chemical vapor synthesis of Ni/graphene nanocomposites with excellent electromagnetic and electrocatalytic properties. Synth. Met..

[24] Wang S.J., Zhang Y.W., Ma H.L., Zhang Q.L., Xu W.G., Peng J., Li J.Q., Yu Z.Z., Zhai M.L. (2013). Ionic-liquid-assisted facile synthesis of silver nanoparticle-reduced graphene oxide hybrids by gamma irradiation. Carbon.

[25] Zhang Q.L., Zhang Y.W., Gao Z.H., Ma H.L., Wang S.J., Peng J., Li J.Q., Zhai M.L. (2013). A facile synthesis of platinum nanoparticle decorated graphene by one-step gamma-ray induced reduction for high rate supercapacitors. J. Mater. Chem. C.

[26] Ma H.L., Zhang L., Zhang Y.W., Wang S.J., Sun C., Yu H.Y., Zeng X.M., Zhai M.L. (2016). Radiation preparation of graphene/carbon nanotubes hybrid fillers for mechanical reinforcement of poly(vinyl alcohol) films. Radiat. Phys. Chem..

[27] Zhang Y.W., Ma H.L., Zhang Q.L., Peng J., Li J.Q., Zhai M.L., Yu Z.Z. (2012). Facile synthesis of well-dispersed graphene by gamma-ray induced reduction of graphene oxide. J. Mater. Chem..

[28] Zhang N., Huang Y., Liu P.B., Ding X., Zong M., Wang M.Y. (2017). Synthesis of magnetical nanoparticles decorated with reduced graphene oxide as an efficient broad band EM wave absorber. J. Alloys Compd..

[29] Zhu Z.T., Sun X., Li G.X., Xue H.R., Guo H., Fan X.L., Pan X.C., He J.P. (2015). Microwave-assisted synthesis of graphene-Ni composites with enhanced microwave absorption properties in Ku-band. J. Magn. Magn. Mater..

[30] Zhao H.T., Li Z.G., Zhang N., Du Y.C., Li S.W., Shao L., Gao D.Y., Han X.J., Xu P. (2014). Gamma-irradiation induced one-step synthesis of electromagnetic functionalized reduced graphene oxide-Ni nanocomposites. RSC Adv..

[31] Huang Y., Ding X., Li S.P., Zhang N., Wang J.G. (2016). Magnetic reduced graphene oxide nanocomposite as an effective electromagnetic wave absorber and its absorbing mechanism. Ceram. Int..

[32] Shi L.L., Zhao Y., Li Y., Han X., Zhang T. (2017). Octahedron Fe_3_O_4_ particles supported on 3D MWCNT/graphene foam: In-situ method and application as a comprehensive microwave absorption material. Appl. Surf. Sci..

